# A New Surgical Technique in Treatment of Nail Onycogryphosis

**Published:** 2018-01

**Authors:** Gholamhossein Ghaffarpour, Zahra Azizian, Mohammad Reza Ghasemi

**Affiliations:** Department of Dermatology, School of Medicine, Iran university of Medical Sciences, Tehran, Iran

**Keywords:** Surgery, Treatment, Nail, Onycogryphosis

**DEAR EDITOR**

Onychogryphosis is an extreme form of irregular, distorted, thickened, hard and discolored, yellow to brown nails on a hyperkeratotic, hyperplastic and onycholytic nail bed usually in the great toe nails of the elderly and infirms. The hypertrophic, twisted and discolored nail plate is shaped like a ram`s horn. Onychogryphosis may rarely occur as a developmental abnormality in young and middle aged and is transmitted as an autosomal dominant trait. Other causes are: ichthyosis, psoriasis, onychomycosis, foot anomaly such as hallux valgus. Treatment of onychogryphosis is either radical or palliative.^1^^,^^2^

Conservative management is especially useful in feet, at high risk patients with peripheral vascular disease and diabetes. Radical treatment consists of surgical removal of the nail and matricectomy by phenolization, Co_2_ laser, etc.^3^ We decided to repair the onychogryphotic toe nail to nearly normal shape by a unique treatment by combination of partial nail matrix phenolization, nail bed widening by longitudinal cuts in the nail bed, onycholysis treatment and displacing the nail matrix and nail bed to opposite direction of nail deviation.

The stages of nail operation were as follows: The patient was prepared by using antibacterial and antiseptic solutions, and 1% povidone iodine. Then anesthesia was carried out with a combination of lidocaine and prelocaine. The great toe was exsanguinated and a flat Penrose drain as a tourniquet was placed on the base of the great toe. The nail plate was separated from dorsal nail fold and lateral nail walls and nail bed using a chisel. A hemostat was used to remove the nail plate from the nail matrix by pulling distally and moving it bilaterally. Two parallel longitudinal cuts were done at the lateral nail folds and a transverse one at the toe tip helping to evaluate the upper and lower surfaces of the nail bed. 

The small particles of the fibrotic tissues on the lower part of the nail bed and the hyperplastic, hyperkratotic and onycholytic tissues were removed in the form of a thin film. In order to expand the nail bed, 3-4 cuts were done longitudinally. For making a smooth surface of the dorsal part of the distal phalynx, it was curated. The hyperplastic nail matrix was phenolized (88.5%) by a cotton swab for 15 seconds. The nail bed was sutured to the lateral nail walls and toe tip by using an absorbable suture (Polyglactine 910=Vicryl 06). In the case of nail deviation to the right or left, the cut at the bottom of the nail bed (phase 6) was extended toward beneath of the nail matrix. The nail matrix as well as the nail bed alignment was sutured the opposite direction of the deviation. 

Therefore, the growth rate of the proximal nail matrix was higher than the distal nail matrix. For this reason, the dorsal surface of the nail plate covered the edge of the lower surface of the nail plate and pushed it downwards and in some case laterally (similar to a couple of metals with different thermal expansion coefficient that are joined with the same length). To modify the difference of this growth rates, phenolization was done for 15 seconds and also in deviated cases, a 5 seconds additional period of phenolization to the opposite direction of the deviation was applied with satisfactory results. At the end, an artificial nail was made of a nasogastric tube and placed on the nail matrix and nail bed and sutured to the both sides. After two weeks, this artificial nail and the stiches were removed and lubricated by an emollient 2-3 times daily until the nail reaches to the toe tip. 

Onychogryphosis was seen in cases that had susceptibility in nail matrix cells to repeat microtrauma, particularly in old ages. It resulted in a thickened, yellow to brown nail plate and distally deviated to right or left. In this process, nail plate was separated from the nail bed (Onycholysis). Onychauxis (nail hypertrophy) was induced by repeated microtrauma to the nail matrix that was hyperplastic without differences in growth rate. So there was no deviation in thickened nail plate when it reached to the fingertip or toe tip. In onychogryphosis, this difference in growth rate lead to deviation to the left or right. The growth rate of the proximal nail matrix was higher than distal nail matrix. Consequently, the dorsal surface of the nail plate passed over the lower surface and pushed the distal end downward. It was similar to a couple of metals with different thermal expansion coefficient that were joined with the same length.

To modify the phenomenon of onychogryphosis, the growth rate of proximal nail matrix decreased. Therefore, phenolization (88.5-90%) was conducted for 15 seconds to help curing the dystrophic nail in several aspects as follows: (i) Denervation of the nerve endings in the base of the nail unit leading to very less or no post operation pain, (ii) A sterile environment was provided at the base of the nail unit. As a result, less prophylactic antibiotic usage was needed, and (iii) A part of proximal nail matrix was destroyed. This lead to a thinner nail plate.^3^

In the cases with nail deviation, the difference of the growth rates was not only in the proximal and distal nail matrix, but also in the sides of proximal nail matrix. For example, if the nail deviated to the right side, the growth rate of the left side was higher than the right side. A 5 sec additional period of phenolization was applied to the left side to control the growth rate of this part. With attention to the deviation and the results, this period was adjusted. In onycholysis induced by onychogryphosis, the nail bed characteristics changed to epidermal structure. For changing the epidermization of nail bed to natural mucous state, the nail bed was detached from the dorsal surface of distal phalynx. Then the fibrosing parts of upper and lower surface of the nail bed were removed. A thin film of hypertrophic, hyperplastic and hyperkeratotic dorsal surface of the nail bed was removed.

In cases of deformation of nail bed to smaller size than normal, 3-4 longitudinal cuts were done to increase the width of the nail bed. The lateral and distal portions of expanded nail bed were sutured to lateral nail walls and distal tip. A split made of a nasogastric tube was placed under the proximal nail fold and on the nail bed to prevent hemorrhage and help the nail bed to have its normal shape and formation and to avoid air exposure. Our findings revealed that if a proper condition for and impaired nail bed is prepared, it can be changed to nail matrix to produce normal nail plate. In more than 90% of the cases this method was done with satisfactory results.
